# Ethical violations and discriminatory behavior in the MedPhys Match

**DOI:** 10.1002/acm2.12135

**Published:** 2017-08-20

**Authors:** Kristi R. G. Hendrickson, Titania Juang, Anna Rodrigues, Jay W. Burmeister

**Affiliations:** ^1^ Department of Radiation Oncology University of Washington Seattle WA USA; ^2^ Department of Radiation Oncology Stanford Cancer Center Stanford CA USA; ^3^ Department of Radiation Oncology Duke University Medical Center Durham NC USA; ^4^ Karmanos Cancer Center Department of Oncology Wayne State University School of Medicine Gershenson Radiation Oncology Center Detroit MI USA

**Keywords:** ethics, medical physics residency, MedPhys match

## Abstract

**Purpose:**

The purpose of this survey study is to investigate behaviors in conflict with the ethical standards of the Medical Physics Residency (MedPhys) Match (MPM) process as stated in the MPM rules (a) and with the nondiscrimination regulations of the Equal Employment Opportunity Commission (EEOC) (b), in addition to other behaviors that may in other ways erode the fairness of the system.

**Methods:**

A survey was sent to all applicants and program directors registered for the 2015 and 2016 MPM. Survey questions asked about application, interview, and postinterview experiences, match results, and overall satisfaction with the process.

**Results:**

Thirteen percent of 2015 respondents and 20% of 2016 respondents were asked by at least one program how highly they planned to rank them or which program they would rank first. Thirty‐seven percent of 2015 and 40% of 2016 program directors indicated that candidates communicated to the program their rank intent, with 22.0% in 2015 and 12.5% in 2016 being told that their program would be ranked first. Twenty‐three percent of 2015 respondents indicated being asked by at least one program during the interview about children or plans to have children; including 19% of males and 33% of females. In 2016, these values were 28% overall, 22% male, and 36% female. Fifty‐seven percent of 2015 respondents who were asked this question indicated being uncomfortable or very uncomfortable answering, including 27.3% of males and 88.9% of females. In 2016, 42.9% of all respondents indicated being uncomfortable or very uncomfortable answering, including 10.0% of males and 80.0% of females.

**Conclusions:**

In the first two years of the MPM, there were widespread instances of ethical violations and discriminatory questioning during the interview process. Educating both interviewers and candidates on the MPM rules and general EEOC guidelines should decrease these instances and increase the fairness of the residency selection process.

## Abstract 2

This article is related content to the editorial: https://doi.org/10.1002/acm2.12169

## 1 INTRODUCTION

The National Resident Matching Program (NRMP) was created in 1952 in an effort to eliminate the chaos, pressure, and gamesmanship that existed when medical residency programs and graduating medical students competed to fill residency slots.^1^, ^2^ The purpose of a residency match system is to provide a fair system to address the problems resulting when many programs compete for the same top candidates and candidates compete for limited residency positions. Without such a system, the recruitment process can result in a race to make the earliest offer, thus pressuring applicants to commit to an early offer that might not be their first choice for fear of not receiving other better offers. Match systems require candidates and programs to submit numerically ranked lists by a single deadline; an algorithm then matches programs with applicants, assigning both their highest available placement rank. However, ethical violations can erode and diminish the potential of a fair match system.^3^, ^4^

Several publications exist in the literature discussing the ethical violations of residency match programs in other medical disciplines, reporting problems with prohibited questioning, postinterview communication, pressures to commit prior to the rank deadline, efforts to game the system, and dishonest communication.^3^, ^5^, ^6^, ^7^ “Gamesmanship” is the term used in the literature to describe manipulating or circumventing the rules and procedures for personal benefit. Some of these publications have a few suggestions for changes, however, the negative aspects of the culture of the residency match system for these disciplines largely did not improve.^8^

Medical physics is a rewarding career for individuals interested in applying their physics background in the medical field. A limited number of CAMPEP‐accredited residency training programs are creating similar levels of competition as experienced in medical residencies.^9^ The MedPhys Match (MPM) began in 2014 with first matching results released in 2015. The pressure on both programs and applicants to successfully match may result in efforts to game the system and potentially the same problematic behaviors that have been documented in other medical disciplines with national matching systems.

A survey of participants in the 2015 and 2016 MPM has been conducted to investigate the occurrence rate of possible behaviors that are in violation of the ethical standards of the MPM, against EEOC guidelines, and general attempts to pressure other parties or to game the system. By conducting this survey in the inaugural and second years of the MPM, a baseline for the culture and conduct of Match participants in Medical Physics is established. By raising this awareness from the beginning, it is hoped that a more positive culture can be established and maintained by the Medical Physics community.

## 2 METHODS

After review by the University of Washington Human Subjects Division that determined the study to be exempt by the Institutional Review Board, a voluntary and anonymous survey was sent to all applicants and program directors registered for the inaugural 2015 MedPhys Match. Initial invitations were sent via email October 9, 2015. One additional reminder was sent 1 week later. The survey was open until October 31, 2015. Each invited participant was sent a unique web link to their respective survey. The survey was conducted by the University of Washington with the consent of the AAPM Medical Physics Residency Training and Promotion Subcommittee. The use of the participants' contact emails for this study was approved by the AAPM Subcommittee on the Oversight of the MedPhys Match. Furthermore, the survey was supported by the AAPM Students and Trainees Subcommittee.

A second year follow‐on survey was sent to all applicants and program directors registered for the 2016 MedPhys Match on June 15, 2016, and open until June 30, 2016. This second survey duplicated the initial survey along with additional questions for program directors comparing the 2 yr of experience and for re‐applicants who did not match in the 2015 MPM and were participating again in the 2016 cycle.

### 2.A The survey instrument

All registered participants (applicants and program directors) were contacted via email. The initial applicants' survey consisted of a total of 57 questions including general demographic information, residency applications, interview experiences, postinterview interactions, MPM experiences and results, and questions regarding their opinion of honesty within the MPM system and their overall experience. At various points throughout the survey, textboxes were provided to encourage additional comments on the MPM experience and suggestions for changes or improvement in the process. The second‐year applicants' survey included a total of 63 questions, adding questions about the previous year's MPM experience for re‐applicants.

The program directors' survey included 35 questions that addressed the numbers of applicants and interviews including changes from previous years, factors that determined or contributed to the program's selection of candidates to interview and rank, the number and mode of interviews (onsite, teleconference, phone), reflections on the interview experience including postinterview communications in general and more specifically related to potential MPM rules violations, opinions on the program's overall MPM experience and current status of the residency/trainee situation. The second year program directors' survey included 36 questions, adding a question regarding the previous year's participation.

Responses to the questions regarding opinions were collected using a 5‐point Likert scale. Study data were collected and managed using REDCap (Research Electronic Data Capture) hosted at the study institution and supported by an institutional grant.^10^ The full survey instruments are included as Supplementary Files.

### 2.B Data analysis

Summary statistics were used to describe the response rates, demographics, interview experiences, ranking and matching experiences, as well as opinions regarding the MPM process. All analyses were conducted using functions available in Excel 2010 (Microsoft, Redmond, WA, USA).

## 3 RESULTS

Applicant surveys were returned by 111 of 402 emailed invitations in the initial survey year, yielding a response rate of 28%, consistent with typical AAPM response rates. One hundred nine surveys were completed and included in this analysis. In the second year survey, 101 of 331 applicants returned the survey, yielding a response rate of 30%. All surveys are included in the analysis.

The demographic distribution of the survey respondents is shown in Table 1. Eighty‐four percent of the respondents submitted a rank list, including 81% of the male respondents and 94% of the female respondents. Forty‐eight percent of the respondents were matched in the inaugural year, including 45% of the male respondents and 58% of the female respondents. This compares with 39% of all applicants who participated in the MPM and were matched (excludes those who withdrew or did not submit ranks).^11^

**Table 1:** Demographic distribution of applicant survey respondents

	2015	2016
Male	73%	64%
Female	32%	33%
Declined to answer	4%	4%
White‐caucasian	64%	51%
Asian	17%	27%
Hispanic‐latino	6%	6%
US Citizen	75%	59%
Foreign citizen	10%	17%
US permanent resident	7%	9%
Canadian citizen	7%	9%
MS		41.6%
PhD		53.5%
Matched	48%	67%
Unmatched	52%	29%
Declined to respond	0	4%

In the second year, 83% of the respondents submitted a rank list, including 81% of the male respondents and 88% of the female respondents. Seventy percent of the respondents matched with a residency position in 2016, including 63% of the male respondents and 87% of the female respondents. This compares to 51% of all applicants participating in the MPM.^11^ Sixty‐eight percent of those respondents who did not match in 2016 indicated that they do intend to apply for residency positions again, and 24% officially withdrew from the MPM before the match deadline. Only 17% of all respondents had also applied to the MPM the previous year, and 65% of those had participated in at least one interview in the previous year.

In the second year survey, 41.6% of respondents indicated a final degree of MS, 53.5% indicated PhD, and 2% did not respond. This question was not included in the first year survey.

**Figure 1:**
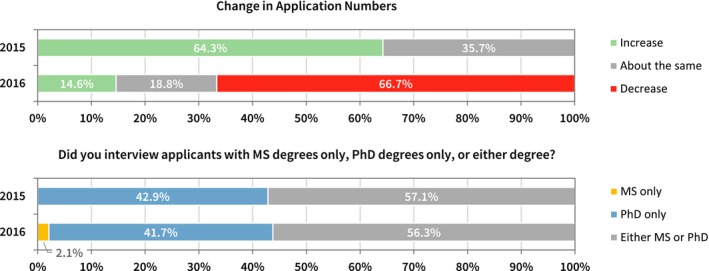
Program directors were asked about changes in application numbers and the education level of the applicants that the programs chose to interview.

Program directors' surveys were returned by 42 out of 79 emailed invitations in the inaugural year, with a response rate of 53%. In the second year survey, 47 surveys were completed in response to 77 emailed invitations, with a response rate of 61%. In the inaugural year survey, none of the responding programs indicated that they interviewed only MS candidates. Forty‐three percent interviewed PhD candidates only, and 57% interviewed both MS and PhD candidates. In the second year survey, one responding program indicated that they interviewed only MS candidates. Forty‐two percent interviewed PhD candidates only, and 56% interviewed both MS and PhD candidates. In the inaugural year survey, program respondents indicated that they largely experienced an increase in applications over the previous year. This situation was reversed in the following year, where most programs reported a decrease in the number of applications (see Fig. 1).

### 3.A The candidate survey: Interview experience

Several questions in the MPM survey asked the candidates about their interview experience, including during and after any interviews in which they participated, to gauge the extent of unethical behavior within the MPM process.

**Figure 2:**
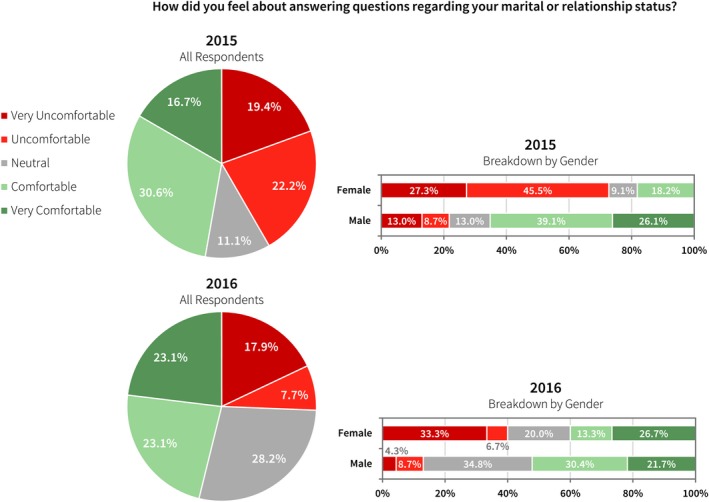
In 2015, 40% of all respondents were asked at least once about their marital or relationship status during their interview. Forty‐two percent were very uncomfortable or uncomfortable answering the question. In 2016, 49% of all respondents were asked this question at least once, including 47% of male respondents and 54% of female respondents.

In the first year survey, 40% of all respondents were asked at least once about their marital or relationship status during their interview. This represented 39% of all male respondents and 41% of all female respondents (Fig. 2). However, female respondents were significantly more uncomfortable answering this question. Of the respondents who were asked about their marital or relationship status, 73% of female respondents were very uncomfortable or uncomfortable answering the question versus 22% of male respondents. In the second year survey, 49% of all respondents were asked about their marital or relationship status, representing 47% of all male respondents and 54% of all female respondents and an increase over first year respondents.

**Figure 3:**
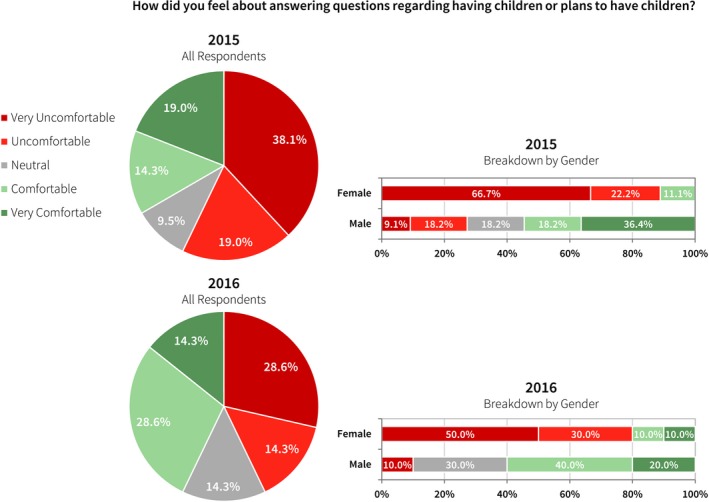
In the first year survey, 23% of all survey respondents indicated that they were asked about having children or their plans to have children during their interview. Of these respondents, 57% were very uncomfortable or uncomfortable answering the question. In the second year survey, 28% of all survey respondents were asked this question. Of the 2016 respondents, 42.9% were uncomfortable or very uncomfortable answering this question, including 10% of the males and 80% of the females.

Twenty‐three percent of all survey respondents in the first year survey indicated that they were asked during their interview about having children or plans to have children. Nineteen percent of all males in the survey were asked this question, and 33% of all females. Of those respondents who were asked this question about children, most were uncomfortable answering the question (57% in 2015 and 42.9% in 2016, see Fig. 3). When the data are separated by gender, female respondents are significantly more uncomfortable answering this question. In the second year survey, 28% of all respondents were asked about children, including 22% of all male respondents and 36% of all female respondents. In 2016 female respondents still overwhelmingly reported general discomfort and males indicated ambivalence in answering this question.

In the first and second year surveys only one and then two survey respondents, respectively, indicated that they were asked about their religion during their interview, representing 1% and 3% of all survey respondents. The respondents indicated that they felt neutral to very comfortable about answering the question.

**Figure 4:**
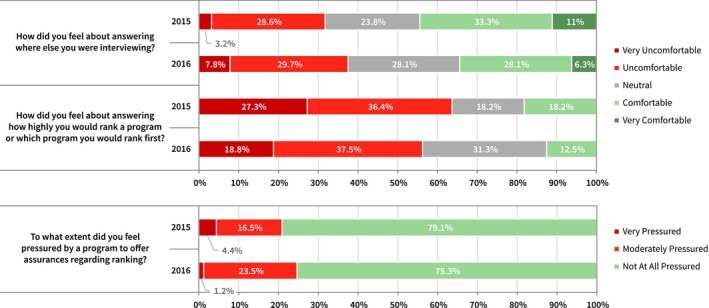
In 2015, 69% of all candidate respondents were asked where else they were interviewing. Of those candidates who were asked, 32% indicated that they were very uncomfortable or uncomfortable answering the question. In 2016, 79% of all respondents were asked this question. In 2015, 13% of all candidate respondents indicated that they were asked at least once how highly they were going to rank the program or asked which program that they would rank number one. In 2016, this value was 20%. The majority of candidates are not comfortable answering this question. Information regarding program ranking may be disclosed during the interview or in postinterview communications. A majority of candidates report not feeling pressured at all to offer assurances to programs regarding ranking.

In the first year survey, 69% of all survey respondents were asked where else they were interviewing. Of those candidates who were asked this question, 32% indicated that they were very uncomfortable or uncomfortable answering the question (see Fig. 4). Twenty‐four percent were neutral in their response, while the remaining respondents were comfortable answering this question. In the second year survey, this value increased to 79% of all survey respondents indicating that they were asked at least once where else they were interviewing; 38% were very uncomfortable or uncomfortable answering this question.

Thirteen percent of all respondents in the first year survey and 16% in the second year survey indicated that they were offered incentives (future faculty position, etc.) during their interview. Twenty‐nine percent in the first year and 27% in the second year surveys indicated that they were told by at least one program that they were “ranked to match” or told their rank number prior to the match deadline. If they had knowledge of their rank position, 78% in the first year and 67% in the second year surveys indicated that the information did *not* affect how they ranked the program. Thirteen percent of all survey respondents in the inaugural year and 20% in the second year surveys indicated that they were asked by at least one program how highly they intended to rank that program or asked which program the interviewee would rank number one. If they were asked about program rank, 64% and then 56% indicated that they were very uncomfortable or uncomfortable answering the question in the 2015 and 2016 surveys, respectively (Fig. 4).

Furthermore, one applicant respondent to the 2015 survey stated, “My only opposition to the process was regarding schools that sent out emails or called multiple people, essentially inferring they were a top pick, only to find out that they had told several other applicants the same thing. This situation happened to several friends going through the process and one friend and I even received nearly identical emails from an institution that was only offering one position.” This respondent continued to complain that this behavior by programs was unfair and misleading to candidates, to the point that applicants altered their rank lists based on the information. This sentiment was echoed in other applicant comments.

Ten percent of all respondents in both years of the survey indicated that they were told by at least one program that they would not match to their program. Seventy‐eight percent of the positive respondents in the inaugural year and 75% in the second year surveys said this knowledge *did* affect how they ranked programs.

Sixteen percent of survey respondents in 2015 and then 15% in 2016 indicated that they were offered a residency position outside the MedPhys Match Program.

### 3.B The candidate survey: Postinterview experience

Fifty percent of all survey respondents in the inaugural year survey indicated that they were contacted via a phone call, email, or letter by a program director, faculty or staff member, or a resident after their interview, which was not in direct response to a contact or question initiated by the candidate. In the second year survey, the positive response rate was 53%. Of all survey respondents, 21% in 2015 and 25% in 2016 said they felt very or moderately pressured by the program to offer assurances (see Fig. 4).

**Figure 5:**
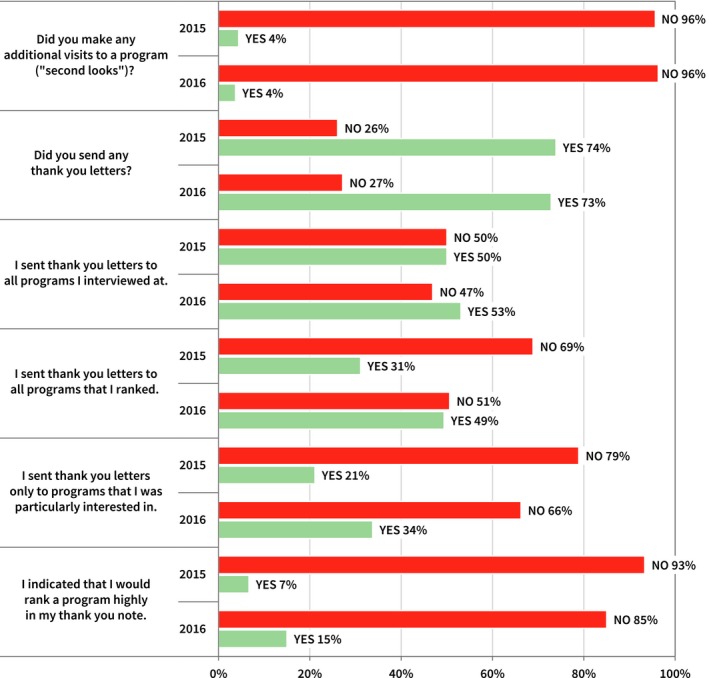
Summary of candidate survey responses regarding thank you letters to the program and disclosure of ranking intent.

Some candidates initiated communication with the program after their interviews. Figure 5 summarizes their responses, where 93% and 85% in the inaugural and second year surveys reported that they did *not* say that they would rank a program highly in their thank you note.

### 3.C Ethical statements

**Figure 6:**
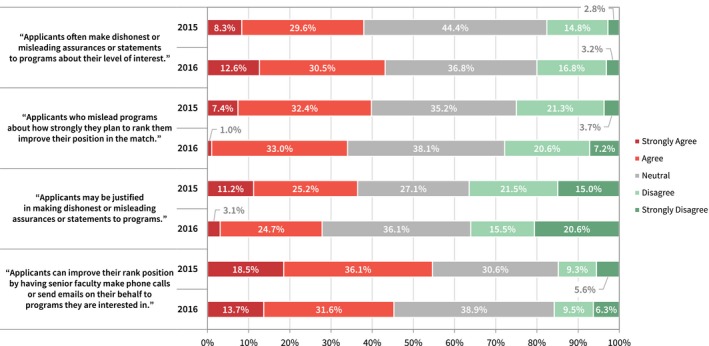
Applicant responses to survey questions related to potential dishonesty among other applicants.

Applicants were asked about their beliefs in the honesty and motivation of participants in the MPM process. In response to “Applicants often make dishonest or misleading assurances or statements to programs about their level of interest,” 37.9% of candidates in the 2015 survey strongly agreed or agreed, while the majority (62.1%) were neutral to strongly disagreed (see Fig. 6). These positive response rates increased in the 2016 survey with 43.1% of candidates indicating that they strongly agree or agree with this statement, while 56.9% were neutral to strongly disagreed.

Candidates were also asked how they felt about the following statement regarding possible results of dishonesty, “Applicants who mislead programs about how strongly they plan to rank them improve their position in the match.” Responses were fairly even among agree, neutral, and disagree in both survey years, as shown in Fig. 6.

As an added value judgment, candidates were asked how they feel about the statement “Applicants may be justified in making dishonest or misleading assurances or statements to programs.” Responses again were fairly even among agree, neutral, and disagree in both surveys, as shown in Fig. 6.

Candidates were asked whether they believed their rank position could be improved if senior faculty from their current institution intervened on their behalf. In the first year survey, the majority (54.6%) responded positively to this question (see Fig. 6). Fewer candidate respondents (45.3%) felt the same way in the second year survey.

### 3.D Match experience

**Figure 7:**
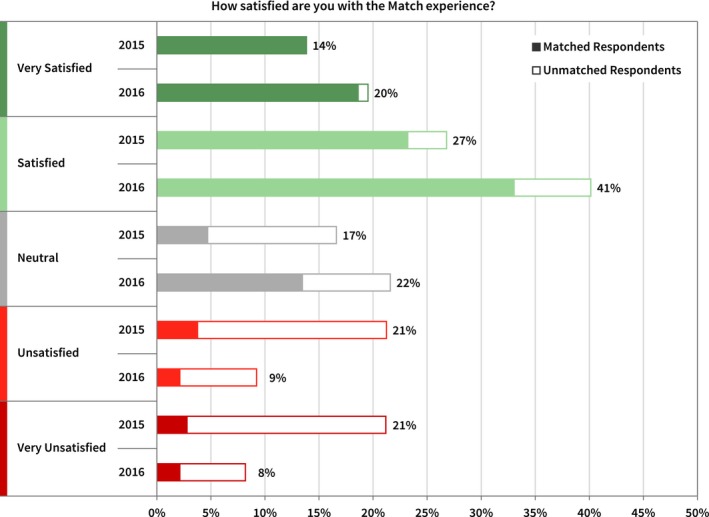
Applicant responses in 2015 and 2016 regarding their overall satisfaction level with the Match experience. The total length of the bar represents the number of respondents in each category, and the solid and open portions of the bar represent matched and unmatched respondents, respectively.

Respondents to the candidate surveys were asked how satisfied they felt with the MPM experience. As shown in Fig. 7, the responses were influenced by whether the individual was matched to a residency, with matched applicants significantly more satisfied with the process overall than applicants left without a residency position.

### 3.E The program directors' survey: Interview experience

Seventy‐seven percent of the program directors who responded to the survey indicated that they did give instructions to resident interview participants on the rules, ethics, and guidelines for Match participation. This positive response rate is consistent with the second year value of 75%.

**Figure 8:**
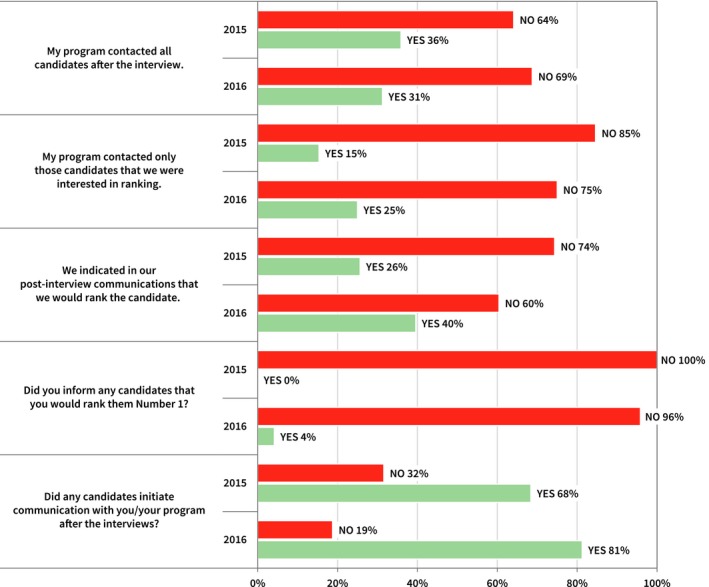
Summary of program directors' postinterview communication with candidates.

Thirty‐one percent indicated that they did initiate some form of communication (phone call, email, or letter) to at least one candidate after their interview that was not in direct response to contact initiated by the candidate. This value is the same in the second year survey. Thirty‐six percent in 2015 indicated that they contacted all candidates after the interview, while 15% indicated that they only contacted those candidates that the program was interested in ranking. In 2016, these values were 31% and 25% respectively. Figure 8 is a summary of the program responses regarding the nature and variety of postinterview communications. None of the program directors responding to the inaugural year survey informed any candidates that they would rank them number one; this value changed to two program directors in 2016.

Sixty‐eight percent of the program directors in the 2015 survey said that candidates initiated communication with the program after the interviews. This value increased to 81% in the second year survey. Thirty‐seven percent and 40% of program directors said candidates communicated their rank intent to the program, in inaugural and second year surveys, respectively. Of these 37% in 2015, none of the programs agreed with the statement that knowledge of the candidate's intent to rank influenced their own ranking of candidates. In 2016, two program directors responded that the intent to rank information *did* influence the program's ranking of candidates. Again of these 37% in 2015, 60% said interviewees indicated that they would rank that program first and 20% were asked by the interviewee how the program would rank them; the values changed to 32% and 0% in the second year survey. Forty‐seven percent of the program directors in both surveys indicated that they felt applicants were (always, frequently, or sometimes) dishonest about their intent to rank the program when the ranking statements were made. Additionally, 13% (two in 2015) and 11% (two in 2016) of the programs failed to match with a candidate that had made a commitment to rank that program number one.

**Figure 9:**
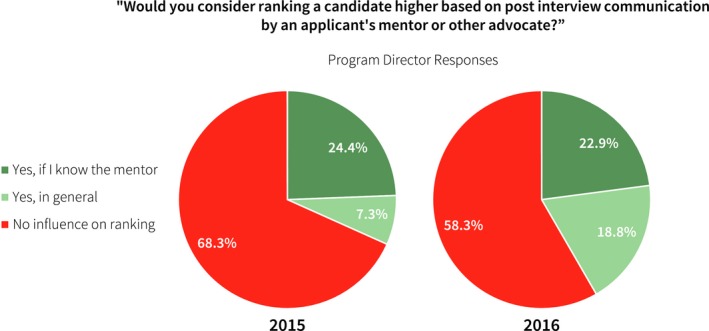
Program directors were asked if communication from the applicant's mentor influenced their ranking decisions. A majority of respondents indicated no influence.

Program directors were asked about the importance or role of postinterview communication by an applicant's mentor or other advocate. In the inaugural year survey, 20% of program directors responded that at least one contact was made on behalf of an applicant. This value decreased to 13% in 2106. Figure 9 shows the relative influence of these communications.

### 3.F Match experience

Ninety‐eight percent (2015) and 92% (2016) of program directors were satisfied or very satisfied with the MPM experience, with 66% and 62.5% very satisfied. In response to a separate question, 25% in 2015 and 23% in 2016 did indicate that the process could be improved, while the majority felt the process is reasonable and needs no changes.

Responses to free text boxes throughout the survey (see File S1 for all survey questions) were deidentified and then shared with the AAPM Subcommittee on the Oversight of MedPhys Match and the AAPM Students and Trainees Subcommittee with the hope that these suggestions might initiate further improvements in the MPM process.

## 4 DISCUSSION

### 4.A Discriminatory questioning

The U.S. Equal Employment Opportunity Commission (EEOC) is responsible for enforcing federal laws that make it illegal to discriminate against a job applicant or an employee because of a person's race, color, religion, gender (including pregnancy), national origin, age, disability, or genetic information. The laws apply to all types of work situations, including hiring and training. The EEOC provides general guidelines for interviewing behavior and a list of inappropriate and/or invasive personal questions that should be avoided.^12^ Basically, any question that could be possibly construed as a reason to discriminate against a candidate should be avoided. These interviewing guidelines should be well‐known in the workplace and are easy to follow.

In this survey, questions about marital status, children or plans to have children, and religion were included. No one should be asked these questions during an interview for residency positions, including all formal and social interactions. It has been previously well‐established, and is clearly supported by Figs. 2 and 3 of this study, that candidates feel they may be discriminated against based on their replies.

Furthermore, one applicant respondent to the 2015 survey commented that *every* program at which she interviewed asked whether she had children or planned to have children during the two‐year residency. These questions left her unsure of how to answer without being dishonest or offending when in fact she knew the line of questioning violated her rights as an interviewee. “My options to answer those questions is [sic] to either say “no” (which may or may not be truthful) or to prefer not to answer which would be taken as a definite yes.”

### 4.B Violations of the letter and spirit of the MedPhys Match rules

The most relevant MedPhys Match rules are contained in The Medical Physics Matching Program Terms of Residency Agreement.^13^ In particular, item 6 states that the program agrees to “require no commitments from applicants and make no offers of appointment to applicants prior to the release of the Match results.” Furthermore, the terms of agreement state the following:

It is understood that during recruitment discussions a program may freely discuss any matter with an applicant, and each may express a high level of interest in the other. Communication guidelines are as follows:

1. A program may voluntarily inform an applicant as to whether or not it intends to rank the applicant
2. An applicant may voluntarily inform a program as to whether or not the applicant intends to rank the program
3. Neither party (program or applicant) may solicit the intentions for ranking from another party
4. Neither party may disclose to the other party or solicit from the other party any information regarding the positioning of any applicant or program on a Rank Order List.

Any expression of interest that may be made during the free discussion between a program and an applicant is subject to change based on further considerations by either party.

The intent is for both applicants and programs to rank their selections in order of their true preference, without regard to how they believe they will be ranked by the other party. That is, to eliminate any “gaming of the system” by pressuring the other party in an effort to ensure a match by making false assurances of rank intent.

Several questions in the candidate and program director's surveys addressed questions explicitly prohibited by the MPM rules (soliciting ranking information and pledging rank order one) and additional questions that–while not explicitly prohibited by the MPM rules–tend to pressure the candidate or program and therefore are inconsistent with the spirit of the rules.

Sixty‐nine percent of applicant respondents in 2015 and 79% in 2016 reported being asked by at least one institution *where else they were interviewing*. Figure 4 shows mixed comfort levels with answering this question. Reasons for the popularity of this question are unclear but could potentially include things such as assessing the candidate's geographic or institutional preferences, the likelihood of matching with them based on the number of interviews they have, or even attempting to gauge a candidate's relative appeal to other residency programs' search committees. None of these are appropriate as the objective of a match system is for each program to rank applicants based on the institution's simple preference among the candidates that they have interviewed. Changes in the rules of the NRMP now explicitly prohibit programs from asking candidates where else they are interviewing, in recognition of reports and discussions in the literature.^14^

Thirteen percent of all respondents in the inaugural year survey and 16% in the second year survey indicated that they were “offered incentives” (future faculty position, etc.) during their interview. Incentives are often meant to pressure the candidate to rank the program highly.

Twenty‐nine percent of all applicant respondents in 2015 and 27% in 2016 reported that they were told by at least one program that they were “ranked to match” or told their rank number prior to the MPM deadline. Thirty‐seven percent and 40% of program directors said candidates communicated their rank intent to the program, in inaugural and second year surveys, respectively. Of these 37%, 60% said interviewees indicated that they would rank that program first and 20% were asked by the interviewee how the program would rank them; the values changed to 32% and 0% in the second year survey. These disclosures of exact rank value are direct violations of match rules, as are questions to the other party of rank intent. Additionally, 13% (2015) and 11% (2016) of the programs failed to match with a candidate that had made a commitment to rank that program number one, which would be impossible to achieve if the candidate did not withdraw from the MPM and indeed had ranked that program number one. Of these 37% in the inaugural year, none of the programs agreed with the statement that knowledge of the candidate's intent to rank influenced their own ranking of candidates, perhaps wisely not taking declared rank intent seriously. In the second year survey, two program directors responded that the intent‐to‐rank information did influence the program's ranking of candidates.

As shown in Fig. 6, candidates are mixed in their perception of whether other candidates are trying to make false claims of rank intent and/or are justified in doing so. It is unclear as to whether candidates feel their chances in the MPM can be enhanced by making rank claims to programs. The survey response by program directors makes it clear that attempts by applicants to game the system or to manipulate the outcome by making false rank claims does not work, in that program directors are skeptical. Forty‐seven percent of the program directors in both surveys indicated that they felt applicants were (always, frequently, or sometimes) dishonest about their intent to rank the program when the ranking statements were made.

While 16% (2015) and 15% (2016) of candidates responded that they were offered a residency position outside of the MPM, we cannot ascertain the nature of these offers. Clearly, it is unethical to use the MPM application and interview process to hire a candidate out of the MPM process (“pair up” with the program and both drop out of the match). We hope that none of the offers identified in this survey represent such an effort to “pair up” and that these were simply positions (faculty, postdoc, or residency) available outside the MPM system that operated on overlapping timelines. Nevertheless, even such fair offers can have undesirable consequences on the MPM process. Indeed, several program directors commented that candidates that they interviewed and ranked withdrew from the MPM before match deadline. Ideally, programs should not have to factor in such attrition when determining the number of applicants to interview and rank. Programs that are hiring during the match recruitment/interview period but not participating in the MPM should therefore be mindful of the effort and expense that programs in the MPM spend on candidates and make every effort to avoid making offers to match participants who have accepted an interview within the MPM until after the match results are released. Such programs are, of course, also encouraged to join the MPM to avoid such difficult situations. Match participants who accept a position outside of the MPM or otherwise withdraw prior to the rank order list deadline are strongly encouraged to immediately contact and inform the residency programs where they have interviewed. The NRMP currently has a rule limiting applicants and programs from discussing, interviewing, or accepting/offering a position outside of their Match between the rank order list deadline and the Monday of Match week.^15^

### 4.C Postinterview communication

Postinterview communications can be merely polite thank you letters from the program to the candidate and/or from the candidate to the program. The communications can also be another opportunity to further influence or pressure the other party by including comments related to rank intentions.

Wu et al. discuss in their 2015 publication that the perceived problem with postinterview thank you notes and other communication such as advocacy calls is that it has been used as a means by which to subtly or overtly convey rank intentions in order to “prearrange” the match ahead of the deadline.^16^ It can take the form of a program promising to rank a candidate highly or “to match” or a candidate claiming that they will rank that program (or every program at which they interview) first in order to convince the other party to do likewise. This behavior is inconsistent with the letter or spirit of residency match rules.

Figures 5 and 8 summarize applicant and program directors responses regarding their use of thank you letters and other postinterview communication. One third of program directors report contacting all candidates after interviews, with 68% (2015) and 81% (2016) reporting that candidates contacted the program after interviews. Program directors reported only contacting those candidates that they were interested in ranking (15% in 2015, 25% in 2016). Three of four candidates reported sending thank you letters to programs, with 21% (2015) and 34% (2016) only sending to programs that they were particularly interested in ranking. Seven percent (2015) and 15% (2016) admitted that they indicated in their thank you letter their intention to rank that program highly. The use of postinterview communication is widespread and clearly some of this communication is used to convey some ranking intention information. In Fig. 4, 79.1% (2015) and 75.3% (2016) of candidates report not feeling pressured at all by programs to offer assurances regarding ranking, which suggests that the use of thank you letters is relatively benign.

### 4.D How does MedPhys Match compare with other medical residency match systems?

Dermatology is an example of a competitive medical field that participates in a national residency matching program with similar rules for ethical conduct. In a 2009 survey study that included Stanford dermatology applicants, current US dermatology residents, and US dermatology program directors, residents felt pressured to reveal rank intent to the programs.^3^ Thirty‐one percent of Stanford applicants and 19% of US dermatology residents responded that they felt pressured to reveal their rank‐ordered lists before the Match deadline. Twenty‐seven percent of program directors reported that they told applicants they were “ranked to match.” Twenty‐one percent of Stanford applicants and 17% of US dermatology residents reported that they were told they were “ranked to match” by the program at which they matched. Fifteen percent of Stanford applicants and 26% of US dermatology residents said they changed their rank‐order lists based on knowledge they received about their rank order from programs. No program director reported promising an applicant an incentive to rank their program highly; however, 5% of Stanford applicants and 3% of US dermatology residents reported that they were promised an incentive to rank a program highly.

Similarly, 21–25% of MPM applicants felt pressured to offer assurances regarding ranking. Twenty‐nine percent (2015) and 27% (2016) reported being told by at least one program that they were “ranked to match” or their rank number prior to the MPM deadline. Furthermore, 13% (2015) and 20% (2016) reported being asked by at least one program how they intended to rank that or other programs.

A follow‐up survey in dermatology was launched the subsequent year, to investigate whether raising awareness of the violations and issues in the initial publication had led to improved behavior.^8^ The conclusions were that the behaviors had not changed.

Jena et al. surveyed senior medical students who had applied to first‐ and second‐year residency positions in the NRMP in a variety of subspecialties.^6^ In this survey, the majority (86.4%) of respondents reported being contacted by at least one residency program after the interview. These communications included feedback suggesting that they would “fit well” in the program (76.2%), that they would be “ranked highly” (52.8%), or that they would be “ranked to match” (34.6%). These types of statements are allowed by NRMP. Five percent of the respondents reported that a program had asked where it would be ranked on the applicant's list; this is a violation of NRMP rules. Sixty‐three percent of the applicants reported that they informed a single program that they would rank it first; while only 1.1% indicated that they informed more than one program that it would be ranked first. Both of those behaviors are violations of the NRMP rules. Almost one‐quarter (23.4%) of respondents reported altering their rank list based on postinterview communications, thereby admitting that they are influenced by the postinterview communication. While only 1.2% of the applicants reported failing to match at a program that had told them that they were “ranked to match” and therefore ranked the program first, 18.6% reported not matching with a program despite feeling assured by postinterview communications that they would match there and therefore ranked the program first. While the nonspecific feedback from programs is allowed by the NRMP rules, it is nonetheless a form of pressure on applicants to alter rankings.

In the MedPhys Match surveys, fewer program directors (36% and 31% in 2015 and 2016, respectively) indicated postinterview communication with candidates compared to the senior medical student survey quoted above. More frequently, communication was initiated by candidates by sending thank you notes to institutions at which they interviewed. According to the survey results, 73% (2015) and 74% (2016) of the candidates responding to the survey sent thank you letters to institutions. Of the program directors who indicated that they received postinterview communication, 37% (2015) and 40% (2016) said candidates communicated their rank intent to the program.

In a 2015 publication by Holliday et al. on the integrity of the Match for Radiation Oncology residency applicants to a single institution, 92.7% reported being asked by a program where else they were interviewing.^7^ The survey was returned by 87 of 171 applicants; including 57 males and 25 females. Sixty‐three percent of all respondents were asked about their marital status during the interview, with 59.7% of males being asked and 72.0% of all females being asked. Twenty‐three percent of all respondents were asked about children or their plans to have children, including 21.1% of the males and 28.0% of the females. Eighteen percent of applicants were told their rank position by the program at which they were interviewing, and 29.3% were asked how they would rank the program. Fifty‐five percent received an unsolicited phone call or email from a program. Half of respondents reported believing that applicants often make dishonest or misleading assurances, with one‐third reporting that they believed their situation improved by deliberately misleading programs. More than two‐thirds reported believing that their rank position could be improved by having faculty contact programs on their behalf.

In a 2016 survey returned by 118 residency applicants to a single radiation oncology department, 84% reported being asked at least once about where else they were interviewing, 51% were asked about marital status, and 22% were asked about plans to have children.^17^ Eighty‐three percent of applicants wrote thank you notes after their interviews, with 55% reporting fear of being viewed unfavorably if they did not. With the widespread report of potential match violations revealed in this survey, 89% stated that they would feel relieved if programs explicitly discouraged postinterview communication.

A survey of 137 obstetrics and gynecology program directors reported that 29% would consider ranking an applicant more favorably if the applicant expressed interest beyond a routine thank you note or if a faculty mentor personally known to the program director endorsed them as outstanding.^18^ Approximately 30% responded that applicants who did not initiate postinterview contact were disadvantaged compared with those who did. The published survey concluded with recommendations for programs to establish and communicate a clear policy to applicants regarding how the program will consider postinterview communications, although it fell short of recommending their elimination.

A survey of urology program directors and resident applicants was completed in 2000 including participation by 230 applicants and 94 program directors.^5^ Forty‐seven percent of program directors recalled being asked by an applicant how the program would rank them; 61% of applicants reported that program directors asked them how they would rank the program. Eighty‐two percent of program directors felt that applicants “lied,” and 67% of applicants felt that programs “lied” about rank intent. Ninety‐one percent of the males and 100% of the females reported being asked about their marital status. Fifty‐three percent of males and 67% of females reported being asked about children. In summary, urology suffers from frequent violations of their match code rules.

### 4.E Study limitations

This study is limited by the overall response rate, and the potential differences in results with a higher response rate cannot be estimated. The survey is confidential, which encourages honest responses. However, there is the added inherent problem in asking for honest answers regarding dishonest behavior that can lead to a potential underestimate of unethical behavior.

This survey study may be biased in that it is a voluntary survey thereby completed only by those sufficiently motivated to follow the supplied link and complete the survey (self‐selection bias). It cannot be known if the responses are representative of all participants in the MPM. In 2015, 48% of applicant survey respondents matched with a residency, and 52% reported not matching (see demographics in Table 1). According to MPM statistics published online, 39% of 2015 applicant participants were matched and 61% not matched.^11^ However, MPM statistics do not include applicants who withdrew, whereas the survey data presented here includes those individuals. It is reasonable to assume all candidates who withdrew according to MPM were in fact unmatched with a residency position (“matching” including only obtaining a residency position through the MPM). MPM statistics then report 27% matched and 73% unmatched. In 2016, survey respondents included 67% matched, 29% not matched, and 4% declined to answer; whereas MPM statistics in that year report 32% matched and 68% not matched (including applicants who withdrew). Therefore there is an over‐representation of survey respondents who matched in 2015 and 2016 compared to the total pool of matched/unmatched applicants.

### 4.F Conclusions and recommendations

Questions about marital or family status and religion are prohibited by EEOC rules and are not meaningful in selecting residents. Casual conversation intended to be harmless could come around to discussing family and related topics. However, programs should clearly instruct their interviewing participants not to initiate any topics related to family, spouse, children, religion, or age. Programs should abstain from asking potentially discriminatory questions. A high percentage of program directors responding to the surveys report that they instruct interview participants on MPM rules and provide guidelines for residency interviews. The content of these instructions is unknown. If general EEOC guidelines have not been included, then they need to be added. All interview participants within the department should be included in this training, not just physics faculty or the search committee.

Competition among residency programs for top candidates and for limited numbers of residency positions can lead to ethical violations when it is believed that promises of high rank and other pressures to commit prior to the MPM deadline are a way for programs to manipulate the system. A few residency positions have been left unfilled at the end of the MPM or were vacated when a matched applicant withdrew legitimately (such as for health reasons) after MPM decisions were finalized. Anecdotally it is known that these positions were filled outside the MPM using lists of unmatched candidates and professional networks with graduate programs.

Other medical specialties tout that their residency positions were filled by their top ranked choices as a measure of their program's value or desirability.^7^ This low “number needed to fill” seems a hollow metric for a relatively small field such as medical physics and therefore is unlikely to be adopted by the medical physics community.

Applicants are under pressure to secure a match given limited residency positions relative to the number of applicants. Programs are at an advantage since they control access to training positions and are generally in a position of power and authority.^3^ However, individual applicants and training programs within a small community such as medical physics do not gain by acquiring a reputation for dishonesty by engaging in unethical behavior in the MedPhys Match system.

Regardless of the potential motivations for unethical or dishonest behaviors within the MedPhys Match, ultimately it is *program* behavior that needs to lead the way by modeling the desired ethical behavior. Suggestions for this model come from the same literature that has reported the ethical violations in other medical residency match programs.^3^, ^4^, ^16^, ^18^, ^19^, ^20^ The recommendations to programs can be summarized as

1. Rank according to quality of the candidate and not on how highly it is believed that they will rank the programs.
2. Do not directly or indirectly divulge ranking intent; and do not solicit this information from candidates.
3. Eliminate postinterview communications to further minimize the risk of divulging or soliciting rank intent information. Questions about the program can be addressed to a neutral and knowledgeable program administrator.
4. Determine rankings immediately following interviews.

Postinterview communication via a thank you letter or by an applicant's mentor has overall little effect on the program's ranking of the candidate. Therefore adopting a system of determining rankings immediately following interviews, such as recommended by Wu,^16^ would have little to no effect on final rankings and makes a clear statement to applicants that any postinterview communication is unnecessary, with or without potentially false promises of ranking intent. This also makes a thank you letter truly a simple, courteous expression of thanks and respect, without the unnecessary and unfortunate need to consider an ulterior motive by the applicant.

Program directors can inform interviewees as a group, “Please feel free to contact us about any questions that you have about our program. We will not ask you how you intend to rank us. We will not share with you where you are positioned in our ranking.” If it is true that the program will determine its rankings immediately following interviews, then it is pertinent and helpful to tell this to all interviewees.

**Figure 10:**
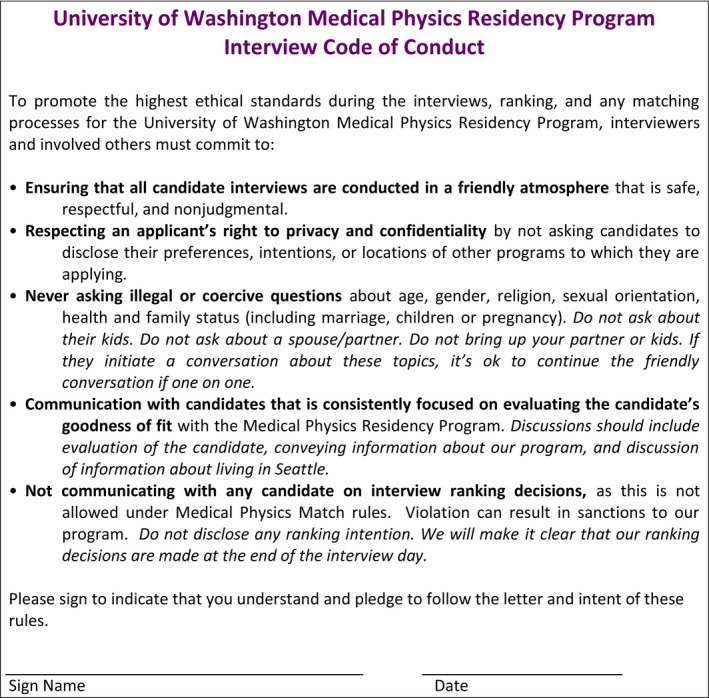
A sample code of conduct agreement statement for all participants in the interviewing process of resident selection.

An example template that programs could choose to adapt and adopt in an effort to educate all interview participants on EEOC guidelines and Match rules is included in Fig. 10.

It is important to note that the AAPM Subcommittee on Oversight of MedPhys Match (SCOMM) is ultimately responsible for investigating any potential Match violations. Potential penalties include a ban from participating in the MPM for a given time period and reporting the violations to other relevant associations such as the Society of Directors of Academic Medical Physics Programs (SDAMPP) or AAPM committees. Comments from program directors in the survey indicated that there is some confusion about what can and cannot be said to applicants and instructions from the MPM were incomplete in this regard, including lack of information on policing of the MPM rules.

## CONFLICTS OF INTEREST

The authors declare no conflicts of interest.

## Supporting information

File S1: All Survey Questions.
