# Supplementary material for: Ethical violations and discriminatory behavior in the MedPhys Match
Source: J Appl Clin Med Phys. 2017 Aug 20;18(5):336–50. doi: 10.1002/acm2.12135 (PMC5874901; doi:10.1002/acm2.12135)
Supplement: Supplementary file 1 — File S1: All Survey Questions. [file ACM2-18-336-s001.docx]

Supplement: All survey questions

**2015 Med Phys Match Survey – Applicants**

Gender (Female/Male/Prefer not to answer)

Ethnicity (American Indian or Native Alaskan/Asian/Black-African American/Hispanic-Latino/Native Hawaiian-Pacific Islander/White-Caucasian/Other/Prefer Not to Answer)

If you answered “Other”, please feel free to specify here (textbox)

Citizenship status (US Citizen/Canadian Citizen/US Permanent Resident/Foreign Citizenship (not US or Canada)/Other/Prefer not to answer)

If you answered “Other”, please feel free to specify here (textbox)

How many residency applications did you submit?

Did you apply to therapy residency programs only? (yes/no)

Did you apply to imaging residency programs only? (yes/no)

Did you apply to both therapy and imaging residency programs? (yes/no)

How many interview invitations did you receive?

Did you decline any interviews you were offered? (yes/no)

If you declined interview offers, what were your reasons for declining? Please rank the importance of each. (Not a reason /Minor reason/Major reason)

Cost of traveling

Scheduling conflict(s) with other interviews

Travel difficulties due to location

Time constraints due to other commitments

Already committed to a sufficient number of interviews

Travel issues due to inclement weather

No longer interested in residency

Other

How many interviews did you attend in person?

How many interviews did you participate in remotely (Skype, other)?

Did you submit a rank list of programs? (yes/no)

What considerations influenced your residency rankings? Please rank the importance of each. (Not a reason /Minor reason/Major reason)

Work environment

Program/Institution reputation

Location

Program structure/organization

Facilities/equipment (e.g., range of treatment modalities, manufacturers)

Feedback from current residents

Program size (larger program preferred)

Program size (smaller program preferred)

Research opportunities

Types of research

Salary versus cost-of-living

Benefits

Other

If "Other", please specify here.

Did you match with a Medical Physics Residency program this year (in 2015)? (yes/no)

If not currently matched, do you intend to apply again? (yes/no)

If no, what alternative are you pursuing? (Medical physics position where residency is not required/Non-medical physics career/ Other/I don't know)

If you answered "Other", please specify here

If yes, how are you preparing for reapplication? (Medical physics research position/Medical physics internship/Medical physics graduate school (additional degree)/Nothing /Other)

If you answered "Other", please specify in the text box.

Did you officially withdraw from the Match? (yes/no)

A residency position was difficult to obtain this past year. (Strongly agree /Agree /Neutral /Disagree /Strongly disagree)

Do you see the current residency placement rate as a problem for our profession? (yes/no)

If I had known the likelihood of getting into a residency program at the time I entered graduate school, then I would have not pursued graduate education in Medical Physics. (Strongly agree/Agree/Neutral/Disagree/Strongly disagree)

The most appropriate place for the filter in the Medical Physics training pipeline is: (Graduate school enrollment/Residency positions/Clinical or other professional positions/Other)

If you answered "Other", please specify.

*Reflections on your interview experience: Thinking back on your interview experiences this year, please report the number of times you encountered the following scenarios (before the match deadline).*

How many times were you asked where else you were interviewing?

If asked, how comfortable were you answering the question? (very uncomfortable/uncomfortable/neutral/comfortable/very comfortable)

How many times were you asked about your marital or relationship status?

If asked, how comfortable were you answering the question? (very uncomfortable/uncomfortable/neutral/comfortable/very comfortable)

How many times were you asked about having children or your plans to have children?

If asked, how comfortable were you answering the question? (very uncomfortable/uncomfortable/neutral/comfortable/very comfortable)

How many times were you asked questions about your religion?

If asked, how comfortable were you answering the question? (very uncomfortable/uncomfortable/neutral/comfortable/very comfortable)

How many times were you were offered incentives (future faculty position, etc)?

How many times were you told by a program that you were "ranked to match" or told your rank number prior to the match deadline?

If you had knowledge of your rank position, did having this knowledge affect how you ranked programs? (yes/no)

How many times were you asked how highly you were going to rank the program or asked which program you would rank number one?

If asked, how comfortable were you answering the question? (very uncomfortable/uncomfortable/neutral/comfortable/very comfortable)

How many times were you told by a program that you would not match to their program?

Did having this knowledge affect how you ranked programs? (yes/no)

How many times were you offered a position outside the MedPhys Match Program?

What is your overall feeling about your interview experiences? (textbox)

Please complete the following yes/no questions regarding communications after interviews.

Did you receive any communications (phone call, email, letter) from a program director/faculty or staff/resident after your interview that was not in direct response to a letter or question from you? (yes/no)

Please rank the extent to which you felt pressured by a program to offer assurances. (very pressured/moderately pressured /not at all pressured)

Did you make any additional visits to a program ("second looks")? (yes/no)

Did you send any thank you letters? (yes/no)

I sent thank you letters to all programs I interviewed at. (yes/no)

I sent thank you letters to all programs that I ranked. (yes/no)

I sent thank you letters only to programs that I was particularly interested in. (yes/no)

I indicated that I would rank a program highly in my thank you note. (yes/no)

Please read the following statements and rate the extent to which you agree or disagree. (strongly agree/agree/neutral /disagree/strongly disagree)

Applicants often make dishonest or misleading assurances or statements to programs about their level of interest.

Applicants who mislead programs about how strongly they plan to rank them improve their position in the match.

Applicants may be justified in making dishonest or misleading assurances or statements to programs.

Applicants can improve their rank position by having senior faculty make phone calls or send emails on their behalf to programs they are interested in.

Please answer the following questions about your overall Match experience.

How satisfied are you with the Match experience? (very satisfied/satisfied/neutral/not satisfied/very unsatisfied)

Which category best represents your total cost of interviewing? (< $500/ $501-1000/ $1001-3000/ $3001-5000/ > $5001)

What is your overall feeling about the Match process? (it is a reasonable process that needs no changes /it could be improved /it is unfair and needs a major overhaul/ it should be discontinued)

Do you have specific suggestions for change in the Match process? (yes/no)

Please specify your suggestions. (textbox)

The following free text box is available for you to communicate any specific concerns or experiences regarding the medical physics residency interview and match process. If you feel you have experienced or witnessed discrimination or a match violation event, please comment here. Reminder: this survey is anonymous and all comments/feedback are appreciated. (textbox)

**2015 Med Phys Match Survey – Program Directors**

How long (years) has your program been accredited?

How long has your residency program been active (including time before and after accreditation)?

How long (years) have you been the Residency Program Director?

Did you have an increase or decrease in the number of applicants to your residency program this year? Comparing before participating in the Match to first year experience in the Match.) (Increase/About the same/Decrease )

Did you interview applicants with MS only, PhD only, or both degrees?

What considerations most influenced your choice of candidates to invite for interview? Please rank the importance of each.

GPA

Graduate program reputation

Research interests

Content/quality of reference letters

Identity of reference letter writers

Medical physics background

Previous clinical experience

Phone interview pre-screening

Personality fit

Other

If you answered "Other", please specify.

What considerations most influenced your final candidate rankings submitted to the match? Please rank the importance of each.

GPA

Graduate program reputation

Research interests

Content/quality of reference letters

Identity of reference letter writers

Medical physics background

Previous clinical experience

Personality fit

Impressions from interview

Other

If "Other", please specify here.

How many residency positions did you offer?

How many of your positions were filled in the match?

How many applicants did you interview onsite?

How many applicants did you interview remotely (phone, Skype, etc)?

Reflections on the interview experience: Thinking back over this year's interviewing season with the match program (2015), please select the response that best reflect your program's experience.

Did you as program director instruct resident interview participants on rules/ethics/guidelines for match participation? (yes/no)

Did you initiate any communications (phone call, email, letter) to a candidate after their interview that was not in direct response to a letter or question from the candidate? (yes/no)

My program contacted all candidates after the interview. (yes/no)

My program contacted only those candidates that we were interested in ranking. (yes/no)

We indicated in our post-interview communications that we would rank the candidate. (yes/no)

Did you inform any candidates that you would rank them Number 1? (yes/no)

Did any candidates initiate communication with you/your program after the interviews? (yes/no)

How many candidates communicated to you/your program their rank intent?

Did this information influence your program's ranking of candidates? (yes/no)

How many times did interviewees indicate that they would rank your program first?

How many times did an interviewee ask you how you would rank them?

Do you feel that applicants were dishonest with you about their intent to rank your program? (Always / Frequently/Sometimes/Never/No applicants revealed their rank intentions to me)

Did you fail to match with any candidate that had made a commitment to you (that is, had communicated that they would rank your program Number 1)? (yes/no)

Would you consider ranking a candidate higher based on post interview communication by an applicant's mentor or other advocate? (Yes if I know the mentor/Yes in general/ No influence on ranking)

How many mentors/advocates for a candidate initiated contact with you/your program to advocate for a candidate?

Please answer the following questions about your overall Match experience.

How satisfied are you with the Match experience? (Very satisfied/ Satisfied/ Neutral/ Not satisfied/ Very unsatisfied)

What is your overall feeling about the Match process? (It is a reasonable process that needs no changes/ It could be improved/ It is unfair and needs a major overhaul/ It should be discontinued)

Do you have specific suggestions for change in the Match process? (yes/no)

Specific suggestions for change. (textbox)

Please answer the following questions about the current status of Residencies in Medical Physics.

There are enough residency positions available to meet the current clinical demand. (Strongly agree/ Agree/ Neutral/ Disagree/ Strongly disagree)

The most appropriate place for the filter in the Medical Physics training pipeline is: (Graduate school enrollment/ Residency positions/ Clinical or other professional positions/ Other)

If you answered "Other", please specify.

Do you see the current residency placement rate as a problem for our profession? (yes/no)

If yes, do you have suggestions for a solution?

The following free text box is available for you to communicate any specific concerns or experiences regarding the medical physics residency interview and match process. If you feel you have experienced or witnessed discrimination or a match violation event, please comment here. Reminder: this survey is anonymous and all comments/feedback are appreciated. (textbox)

**2016 Med Phys Match Survey – Applicants**

Survey questions same from 2015 with the following additional questions added in 2016:

Please specify your related education (completed or in progress with completion imminent) (MS Medical Physics/ PhD Medical Physics/ PhD Other / CAMPEP certificate)

Did you apply for a residency through MedPhys Match last year (applied in 2014 for 2015 residency)? (yes/no)

Did you participate in at least one residency interview last year (in 2015)? (yes/no)

Did you submit a rank list in the previous year ? (yes/no)

What experiences did you pursue in the year between the match cycles? Choose all that apply. (Additional medical physics education/ Medical physics research (post-doc, other)/ Non-medical physics research (post-doc, other) / Medical physics employment / Non-medical physics employment / Volunteer clinical medical physics work / Industry / Other)

If you answered "Other," please specify here

Please comment on how your match experience compares in 2016 to your previous experience in 2015. (textbox)

**2016 Med Phys Match Survey – Program Directors**

Survey questions same from 2015 with the following additional questions added in 2016:

Did you participate in the MedPhys Match last year as well?

What considerations most influenced your choice of candidates to invite for interview? Please rank the importance of each.

GPA

Graduate program reputation

Research interests

Content/quality of reference letters

Identity of reference letter writers

Medical physics background

Previous clinical experience

Phone interview pre-screening

Personality fit

*Academic potential*

*Leadership potential*

*Clinical potential*

Other

If you answered "Other", please specify.

What considerations most influenced your final candidate rankings submitted to the match? Please rank the importance of each.

GPA

Graduate program reputation

Research interests

Content/quality of reference letters

Identity of reference letter writers

Medical physics background

Previous clinical experience

Personality fit

*Seminar presentation*

Impressions from interview

*Academic potential*

*Leadership potential*

*Clinical potential*

Other

If "Other", please specify here.
